# Genetic Relatedness of Dengue Viruses in Key West, Florida, USA, 2009–2010

**DOI:** 10.3201/eid1904.121295

**Published:** 2013-04

**Authors:** Jorge L. Munoz Jordan, Gilberto A. Santiago, Harold Margolis, Lillian Stark

**Affiliations:** Centers for Disease Control and Prevention, San Juan, Puerto Rico (J.L. Munoz Jordan, G.A. Santiago, H. Margolis);; Florida Department of Health, Tampa, Florida, USA (L. Stark)

**Keywords:** dengue virus, DENV-1, Key West, Florida, phylogeny, viruses, vector-borne infections

## Abstract

Sequencing of dengue virus type 1 (DENV-1) strains isolated in Key West/Monroe County, Florida, indicate endemic transmission for >2 years of a distinct and predominant sublineage of the American–African genotype. DENV-1 strains isolated elsewhere in Florida grouped within a separate Central American lineage. Findings indicate endemic transmission of DENV into the continental United States.

Dengue is the most common mosquito-borne viral disease; cases have been reported from ≈100 countries, and there are indications of increased incidence and severity worldwide (*1*). The United States has reported year-round transmission of dengue virus (DENV) in Puerto Rico, the US Virgin Islands, and American Samoa and occasional transmission along the Texas–Mexico border. In the continental United States, DENV is the most frequent cause of febrile illness among travelers returning from the Caribbean, South America, and Asia (*2*,*3*). These frequent introductions of dengue infections and the increased presence of vectors (i.e., *Aedes aegypti* and *Ae.* *albopictus* mosquitoes) in many US regions may portend the reintroduction and extended transmission of DENV into the continental United States.

In September 2009, the Florida Department of Health (FDOH) and the Centers for Disease Control and Prevention (San Juan, Puerto Rico) investigated a case of DENV type 1 (DENV-1) infection in a person (index patient) who, as confirmed by reverse transcription PCR (RT-PCR), acquired the virus while traveling to Key West in Monroe County, Florida, USA. DENV-1 infections were subsequently confirmed in 2 Monroe County residents without histories of recent travel. In addition, among 13 other cases in the county that were identified by serologic methods, 2 were confirmed as DENV-1 infections (*4*). Thus, a total of 5 DENV-1 cases were confirmed in Key West during 2009, and ≈5% of the serosurveyed population in Key West had evidence of recent DENV infection (*4*,*5*). In 2010, additional dengue cases from Monroe County were reported, and DENV-1 was isolated from a mosquito pool (*6*) and a blood donor from Key West (*7*); isolates from the mosquito pool and blood donor appeared to be phylogenetically related (*7*). This study determined the genetic relatedness of the DENV-1 isolates from dengue patients in Key West and 4 other Florida counties during 2009–2010, including the blood donor and mosquito isolates.

## The Study

During 2009–2010, serum samples from patients with suspected dengue were received by the FDOH for dengue diagnostic testing; the samples came from 16 of Florida’s 67counties. All samples were tested by using DENV serotype–specific, real-time RT-PCR (*8*) and IgM anti-DENV ELISA (*9*). Samples with highly positive RT-PCR results were spread onto cultured *Ae*. *albopictus* C6/36 cells, and the presence of virus and genome were confirmed by immunofluorescence (*10*) and RT-PCR, respectively (*11*). Isolates were further propagated and viral RNA was extracted from culture supernatants by using the Universal BioRobot System (QIAGEN, Valencia, CA, USA). The envelope glycoprotein (*E*) gene was amplified (Technical Appendix), and the *E* gene open-reading frame (1,485 bp) was sequenced. All sequences were submitted to GenBank; accession numbers are shown in Technical Appendix. Multiple sequence alignments were performed by using the MUSCLE module available in MEGA 5 (www.megasoftware.net). Evolutionary history was inferred by using maximum likelihood and phylogenetic trees constructed by using neighbor-joining methods. Evolutionary distances were computed, and several *E* gene sequences from GenBank were included in the phylogenetic tree to support tree topology by genotype (Technical Appendix).

In 2009, five DENV-1–positive cases were identified by RT-PCR in Key West. Subsequently, in 2010, the FDOH tested 195 serum samples by real-time RT-PCR. Fifty-six (29%) samples were positive for DENV RNA: DENV-1 (37 [66%] samples), DENV-2 (13 [23%] samples), DENV-3 (3 [5%] samples), and DENV-4 (3 [5%] samples). Monroe County submitted 73 serum samples, of which 31 (42%) had results positive for recent dengue infection: DENV-1 was detected in 22 by RT-PCR, and 9 had positive IgM anti-DENV ELISA results. No other DENV serotype was identified in Key West. None of the DENV-1 patients from Monroe County had a history of recent travel to a dengue-endemic region before the onset of symptoms. Fifteen other Florida counties submitted serum samples: 13 counties submitted <10 specimens, 1 submitted 20–30 specimens, and 1 submitted >60specimens. DENV-1 was found in 15 serum samples from 6 of these counties; however, all the patients had a history of recent international travel.

**Figure 1:**
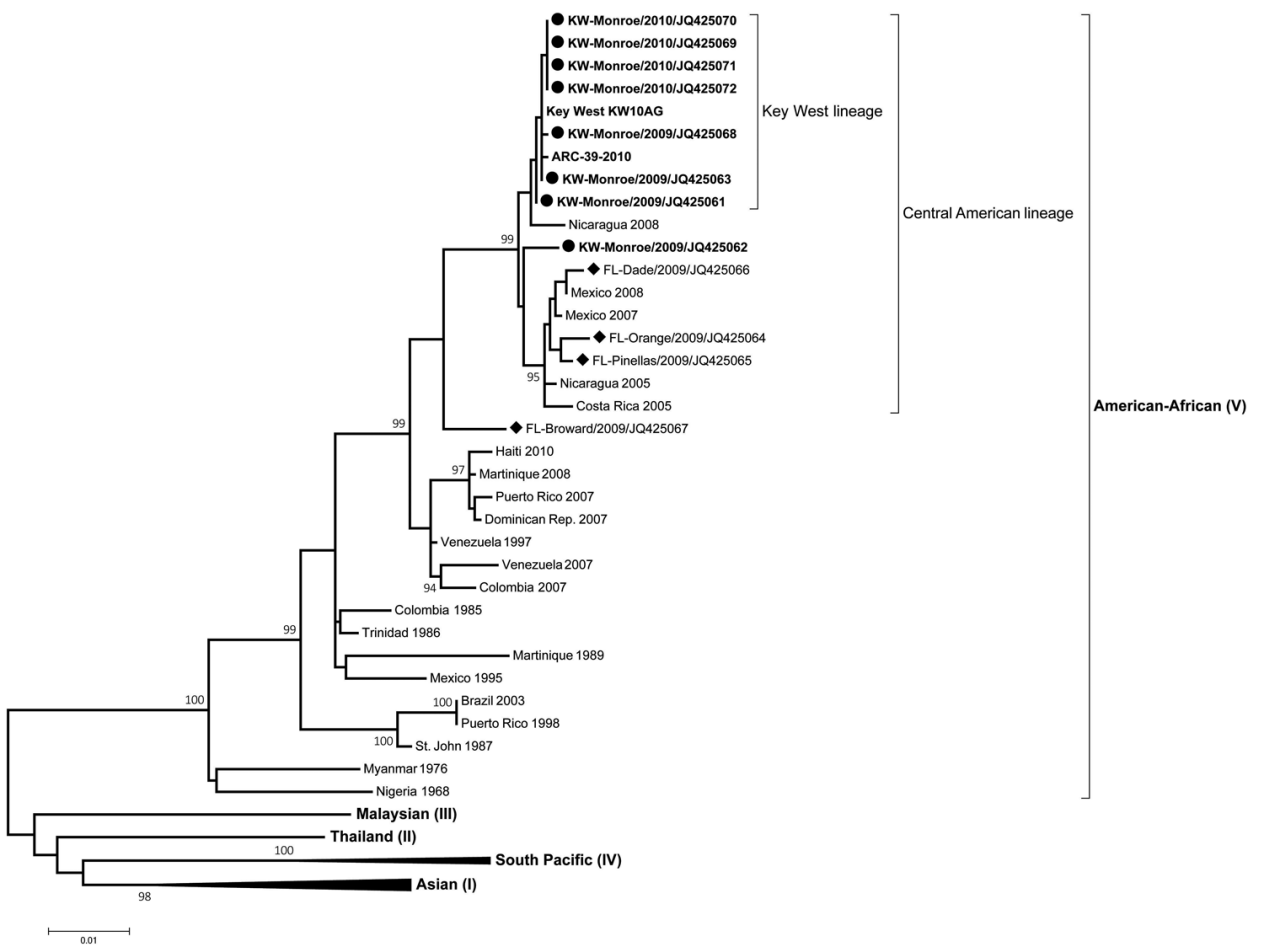
Maximum-likelihood phylogenetic tree of dengue virus type 1, including isolates from Key West, Florida, USA, and representative isolates from 5 genotypes with global geographic distribution. Solid circles, 8 Key West viruses (Monroe County) isolated during 2009–2010; solid diamonds, isolates from other Florida counties (Dade, Pinellas, Orange, and Broward Counties). Scale bar indicates nucleotide substitutions per site. Each taxon represents a single virus isolate and is labeled with the geographic origin and collection year. All Florida viruses were labeled with the county of origin. **Boldface** taxa labels indicate the Key West lineage and cases not associated with travel. Isolate KW-Monroe/2009/JQ425068 represents the 2009 Key West outbreak index case virus. Thirty-six envelope glycoprotein gene sequences obtained from GenBank were included to support tree topology and identify genotypes. All genotypes except the American–African genotype (V) have been collapsed. Taxa labels and GenBank accession numbers are available in Technical Appendix.

We sequenced the *E* gene of 12 DENV-1 strains isolated in Florida during 2009–2010 to determine their genetic relatedness; of the 12 strains, 8 were from Key West and 1 each was from Dade, Broward, Orange, and Pinellas Counties. In addition, 23 DENV-1 *E* sequences published in GenBank, including the 2010 Key West isolates obtained from a blood donor and a mosquito pool (*6*,*7*), were used to construct a maximum-likelihood phylogenetic tree. The significance of branch lengths and taxa relationships was tested by 1,000 bootstrap replications. This phylogenetic analysis showed that all the Florida DENV-1 isolates belong to the American–African genotype (genotype V) (*12*,*13*) together with other viruses isolated throughout the Americas (Figure 1). 

Key West DENV-1 viruses grouped among Central American viruses, which configure a distinct lineage separate from the Caribbean viruses. This divergence between the Central American and Caribbean lineages is well supported by high bootstrap values. Moreover, the Key West and Monroe County viruses grouped together and indicated a distinct sublineage supported by a high bootstrap value (99%), separating them from viruses isolated in Dade, Orange, Pinellas, and Broward Counties that were more closely related to other Central American viruses (Figure 1). One 2009 isolate (IQ425062) from a Key West patient is related to this group, suggesting a separate introduction of DENV-1 in Key West. The sequence similarity between the 2009 and 2010 Key West strains was <0.9%; however, the evolutionary distance and taxa positions between the 2009 and 2010 strains presented in the phylogenetic tree suggests that the 2010 strains diverged from the 2009 strains. The observed differences between *E* gene sequences for the Key West strains (2009–2010) and the rest of the strains in this phylogeny were <2.1% with the other Florida strains, <1.2% with Central American strains, and <4.8% with the rest of the American–African genotype.

## Conclusions

Evolutionary distances and the topology of the Central American lineage suggest this lineage is the genetic origin of the Florida DENV-1 strain. Most viruses isolated in Monroe County diverged from the Central American lineage into a distinct sublineage—the Key West DENV-1 strain associated with the 2009–2010 outbreak. The high level of genetic similarity among the viruses isolated in Monroe County, their close evolutionary distances, and the lack of recent international travel for the case-patients suggest endemic transmission and microevolution of this DENV. Conversely, the scattered and separate phylogenetic positioning of virus strains from patients with travel-associated cases from other Florida counties indicates a different origin from the majority of Key West isolates. Although the 2009 Broward isolate (JQ425067) is positioned near the Central American lineage, the low bootstrap value (53%) does not support lineage ancestry.

The epidemiologic and phylogenetic evidence suggests that the 2010 cases appeared to be a continuation of the 2009 outbreak. Unlike cases along the Texas–Mexico border (*14*), all DENV-1 infections in Key West seem to have been locally acquired. *Ae*. *aegypti* mosquitoes collected by the FDOH were positive for DENV-1. In addition, DENV-1 was detected in a blood donation from the Monroe County in 2010, further supporting local transmission of DENV (*7*). Collectively, these findings indicate that endemic DENV-1 was transmitted in Key West over a period of >2 years.

Technical Appendix Table showing the dengue virus type 1 (DENV-1) envelope gene reverse transcription PCR and another showing DENV-1 strains used in a study of the genetic relatedness of dengue viruses in Key West, Florida, USA, 2009–2010.
