# Supplementary material for: Genetic Relatedness of Dengue Viruses in Key West, Florida, USA, 2009–2010
Source: Emerg Infect Dis. 2013 Apr;19(4):652–4. doi: 10.3201/eid1904.121295 (PMC3647415; doi:10.3201/eid1904.121295)
Supplement: Technical Appendix — Table showing the dengue virus type 1 (DENV-1) envelope gene reverse transcription PCR and another showing DENV-1 strains used in a study of the genetic relatedness of dengue viruses in Key West, Florida, USA, 2009–2010. [file 12-1295-Techapp-s1.pdf]

Publisher: CDC; Journal: Emerging Infectious Diseases
Article Type: Dispatch; Volume: 19; Issue: 4; Year: 2013; Article ID: 12-1295
DOI: 10.3201/eid1904.121295; TOC Head: Dispatch

Article DOI: http://dx.doi.org/10.3201/eid1904.121295

# Genetic Relatedness of Dengue Viruses in Key West, Florida, USA, 2009–2010

## Technical Appendix

Table 1. Dengue virus type I envelope gene reverse transcription PCR primers used in a study of the genetic relatedness of dengue viruses in Key West, Florida, USA, 2009–2010

| Primer name | Sequence | Position |
|---|---|---|
| D1–1 | AGTTGTTAGTCTACGTGGACCGAC | 1–24 |
| cD1–2086 | CACCTGCTCCTACCACGATGTAGC | 2065–2085 |
| D1–1880 | CAGCATGGAACTGTTCTGGTGCAG | 1881–1904 |
| cD1–4196 | GAGGAGTGAACTTAGTAGGATGCT | 4173–4196 |

Table 2. Dengue virus type I strains used in a study of the genetic relatedness of dengue viruses in Key West, Florida, USA, 2009–2010

| Taxa label | Geography | Collection year | GenBank accession no. |
|---|---|---|---|
| ARC-39–2010 | Key West, Florida, USA | 2010 | JQ045564 |
| Brazil 2003 | Brazil | 2003 | JF804014 |
| People’s Republic of China 1980 | People’s Republic of China | 1980 | AF350498 |
| Colombia 1985 | Colombia | 1985 | AF425616 |
| Colombia 2007 | Colombia | 2007 | JF804015 |
| Costa Rica 2005 | Costa Rica | 2005 | JF804016 |
| Dominican Rep. 2007 | Dominican Republic | 2007 | JF804017 |
| Haiti 2010 | Haiti | 2010 | JF969282 |
| Hawaii 1944 | Hawaii | 1944 | AB609588 |
| Indonesia 1988 | Indonesia | 1988 | AB074761 |
| Key West KW10AG | Key West, Florida, USA | 2010 | JF519855 |
| KW-Monroe/2009* | Key West, Florida, USA | 2009 | JQ425061 |
| KW-Monroe/2009* | Key West, Florida, USA | 2009 | JQ425062 |
| KW-Monroe/2009* | Key West, Florida, USA | 2009 | JQ425063 |
| KW-Monroe/2009* | Key West, Florida, USA | 2009 | JQ425068 |
| KW-Monroe/2010* | Key West, Florida, USA | 2010 | JQ425069 |
| KW-Monroe/2010* | Key West, Florida, USA | 2010 | JQ425070 |
| KW-Monroe/2010* | Key West, Florida, USA | 2010 | JQ425071 |
| KW-Monroe/2010* | Key West, Florida, USA | 2010 | JQ425072 |
| FL-Orange/2009* | Orange County, Florida, USA | 2009 | JQ425064 |
| FL-Pinellas/2009* | Pinellas County, Florida, USA | 2009 | JQ425065 |
| FL-Broward/2009* | Broward County, Florida, USA | 2009 | JQ425067 |
| FL-Dade/2009* | Dade County, Florida, USA | 2009 | JQ425066 |
| Malaysia 1972 | Malaysia | 1972 | AF425622 |
| Martinique 1989 | Martinique | 1989 | JF804018 |
| Mexico 1995 | Mexico | 1995 | DQ341194 |
| Mexico 2007 | Mexico | 2007 | HQ166036 |
| Mexico 2008 | Mexico | 2008 | GU131965 |
| Myanmar 1976 | Myanmar | 1976 | AF425615 |
| Nicaragua 2005 | Nicaragua | 2005 | FJ850113 |
| Nicaragua 2008 | Nicaragua | 2008 | GQ199858 |
| Nigeria 1968 | Nigeria | 1968 | AF425625 |
| Philippines 2004 | Philippines | 2004 | JF804020 |
| Puerto Rico 1998 | Puerto Rico | 1998 | JF804021 |
| Singapore 2008 | Singapore | 2008 | JN022600 |
| St. John 1987 | St. John, US Virgin Islands | 1987 | JF804023 |
| Tahiti 2001 | Tahiti | 2001 | JF804024 |

| | | | |
|---|---|---|---|
| Thailand 1964 | Thailand | 1964 | AF180817 |
| Thailand 1997 | Thailand | 1997 | JF804025 |
| Thailand 2004 | Thailand | 2004 | JF812100 |
| Thailand 2006 | Thailand | 2006 | JF812097 |
| Thailand 1980 | Thailand | 1980 | AF425630 |
| Trinidad 1986 | Trinidad and Tobago | 1986 | AF425639 |
| Venezuela 1997 | Venezuela | 1997 | AF425634 |
| Venezuela 2007 | Venezuela | 2007 | GU131840 |
| WestPac 1974 | Nauru | 1974 | M23027 |

*Virus isolates sequenced for this study
